# Biological shifts of endemic pearl mullet (*Alburnus tarichi*) during spawning migration between a highly alkaline soda lake and a river

**DOI:** 10.1007/s10695-026-01672-6

**Published:** 2026-03-30

**Authors:** Esin G. Canli, Elif Kaval Oguz, Ahmet R. Oguz, Mustafa Canli

**Affiliations:** 1 Department of Biology, Faculty of Sciences and Letters, Cukurova University, Adana, Turkey; 2 Science Education, Faculty of Education, Van Yuzuncu Yil University, Van, Turkey; 3 Department of Biology, Faculty of Science, Van Yuzuncu Yil University, Van, Turkey

**Keywords:** Antioxidant, Osmoregulation, Anadromous migration, Adaptation, Histology

## Abstract

Lake Van is the largest lake in Türkiye (3.775 km^2^) and the world’s largest soda lake. Pearl mullet (*Alburnus tarichi*), also known as the Van fish, is endemic to Lake Van and is accepted as an endangered species. Pearl mullet migrate from Lake Van to the freshwater streams in spring for spawning. This study aimed to investigate the responses of the antioxidant, osmoregulation, and energy metabolism and also the histological changes in pearl mullets before and after spawning. Fish were sampled in the mouth of the River Karasu for the pre-spawning and the upper part of the river for post-spawning. The size of fish (5 male and 5 female) did not differ significantly (*p* > 0.05) between samplings. Na⁺, K⁺-ATPase, Mg-ATPase, and Ca-ATPase activities in post-spawning samples were significantly (*p* < 0.05) higher in the osmoregulatory organs (gill or kidney). Likewise, activities of antioxidant enzymes in the liver increased (*p* < 0.05) after spawning. Oppositely, Fe levels in the kidney were much higher (*p* < 0.05) in pre-spawning compared to post-spawning. However, acetylcholinesterase activity in the muscle and brain did not change significantly (*p* > 0.05) during the migration. Heat shock protein (HSP70 +) cells in tissues increased in fish after spawning. The cost of metabolic shift was reflected in the immediate energy reserves of the muscle and liver, as the levels declined significantly (*p* < 0.05) after spawning. Additionally, histological changes were observed in the gills and liver during spawning migration, but not in the digestive tract. This study presented comprehensive data on the biological adaptation mechanisms of pearl mullet during anadromous migration, emphasising its vulnerability.

**Graphical Abstract:**

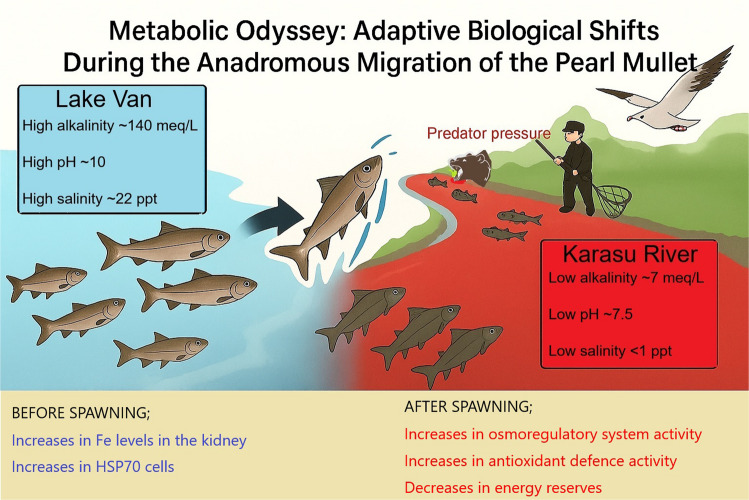

## Introduction

Lake Van has extreme environmental conditions, such as high pH (9.8), salinity (21.4‰), and alkalinity (153 meq/L). The lake severely restricts aquatic life (Danulat 1995). Despite it being the largest (3.775 km^2^) lake in Türkiye, only a few organisms inhabit the lake, such as certain plankton species, chironomid larvae, and pearl mullet (*Alburnus tarichi*). Being an anadromous species, it undertakes large-scale spawning migrations into freshwater streams. While the timing of this reproductive migration varies with seasonal temperatures, it generally occurs between April and July. Following reproduction, the fish can return directly to the lake. During their time in freshwater before spawning, the fish are exposed to various stressors, including high population density, predators (birds, snakes, and humans), fasting, microbial and parasitic infections, and intense energy expenditure due to swimming against the current (Oguz and Kaval Oguz, 2020). Additionally, the metabolic changes and reproductive success observed during these migrations are critical to the species’ sustainability. Considering the endemic nature of this species and its “endangered” status on the IUCN Red List (Freyhof 2014), it is essential to investigate the physiological, ecological, and anthropogenic factors influencing it.

The importance of gill and kidney tissues in osmoregulatory processes is well established, especially in migratory fish transitioning between waters with different chemical compositions (e.g. anadromous and catadromous species). Ion-ATPase group enzymes, particularly Na⁺/K⁺-ATPase, play a crucial role in osmoregulatory adaptation by actively transporting sodium and potassium ions against their concentration gradients. Specifically, Na⁺/K⁺-ATPase exports 3 Na⁺ ions out of the cell while importing 2 K⁺ ions to maintain the electrochemical balance. All ATPases use ATP energy to transport ions. It is estimated that ATPases consume approximately 20% of the ATP produced in animal cells, and this can reach 66% in neurons (Howarth et al. 2012). Similarly, Mg-ATPase is an ATP-dependent enzyme that regulates Mg^2^⁺ ions during oxidative phosphorylation in mitochondria and also maintains intracellular Mg^2^⁺ levels outside mitochondria. Two isoforms of Mg-ATPase are known: one located in mitochondria and the other predominantly found in the endoplasmic reticulum (Canli & Stagg 1996). In addition to Mg-ATPase, another important ATPase is Ca-ATPase, which plays a critical role during muscle contraction by maintaining low intracellular Ca^2^⁺ levels through active transport across the sarcoplasmic reticulum. This transport process is also ATP-dependent and entails significant energy consumption.

Acetylcholinesterase (AChE) is a key enzyme of the cholinesterase family that facilitates the transmission of nerve impulses by hydrolysing the neurotransmitter acetylcholine into acetate and choline. Importantly, neuromuscular function is dependent on AChE activity, as any dysfunction in this enzyme may lead to impaired locomotion. The connection between neurons and muscles operates effectively only when AChE swiftly removes acetylcholine from the synaptic cleft (Silva-Herdade and Saldanha 2013; Canli et al. 2019). Given the intense locomotor demands during spawning migration, AChE activity may undergo substantial changes. Furthermore, AChE activity could be altered by environmental pollutants or stress (Rao et al. 2005).

Animal metabolism consumes substantial amounts of energy under conditions that demand high levels of activity, such as migration, as well as during various stressful circumstances (Bossuyt and Janssen 2004). The primary sources of chemical energy stored as ATP are carbohydrates, lipids, and proteins, with carbohydrates (primarily glucose) serving as the initial energy source. However, under conditions requiring excessive energy, lipids and proteins may also be utilised sequentially as alternative energy sources. In this context, since the liver and muscle tissues are the main sites where energy sources are stored and metabolised, investigations of energy metabolism in these tissues can provide meaningful insights (Bossuyt and Janssen 2004; Canli 2005). In migratory fish species such as the pearl mullet, energy demands may increase due to significant changes in osmoregulation and associated stress. Disruptions in energy metabolism may affect general physiological functions, ultimately threatening the survival of these fish.

Animal cells strive to cope with oxidative stress originating from internal and external sources, and the antioxidant defence system is used to reduce the oxidative stress. There are both enzymatic and non-enzymatic molecules that work together to mitigate oxidative stress. The enzymes involved in this system can be summarised as follows: Superoxide dismutase (SOD) converts superoxide anion radicals into hydrogen peroxide. Catalase (CAT) reduces hydrogen peroxide into water. Glutathione peroxidase (GPX) eliminates hydrogen peroxide and other peroxides as well as superoxide radicals and converts oxidised glutathione into its reduced form. Glutathione S-transferase (GST) catalyses the conjugation of GSH with xenobiotic compounds, while glutathione reductase (GR) reduces oxidised glutathione back to GSH, using it as an electron donor. There is a natural balance between free radicals and antioxidant defence systems in aerobic organisms. When this balance shifts in favour of free radicals, oxidative stress occurs (Winston 1991). In this context, antioxidant enzymes play a critical role in coping with oxidative stress induced by environmental factors (Canli et al. 2023). Among these, glutathione (GSH) is a tripeptide found in a wide range of cells from microorganisms to humans. Although GSH is produced in all organs, it is synthesised most abundantly in the liver. As a non-protein thiol with low molecular weight, GSH is a crucial antioxidant found in high concentrations in the intracellular fluid (Meister and Anderson 1983). The sulfhydryl (-SH) group of the cysteine residue in GSH is its most active site, participating in reduction and conjugation reactions to detoxify xenobiotics and peroxides (Meister 1992). Because these detoxification processes are closely linked with oxidative stress, studying the oxidative stress metabolism may provide crucial insights into the stress physiology of fish, as they face high levels of stress during their spawning migration.

Heat shock proteins (HSPs) are a family of proteins that help prevent cellular damage. They are found in all organisms and are classified based on their molecular weight. The most studied HSPs include HSP60, HSP70, and HSP90. These proteins play essential roles under internal and external stress conditions. In aquatic organisms, HSPs have been reported to be expressed in various tissues in response to environmental and physiological stress (Iwama et al. 1998; Rajeshkumar et al. 2013). They are synthesised by cells in response to a range of biotic and abiotic stressors, such as extreme temperatures, parasitic infections, and pollutants (Iwama et al. 1998). Therefore, understanding the responses of the antioxidant defense system and HSP family proteins during migration could yield valuable insights into fish metabolism. Several studies have investigated different aspects of spawning migration in *A. tarichi*, including gonadal development (Unal et al. 2007), histological changes (Oguz 2015; Çelebi & Oğuz 2020; Alkan & Oğuz 2021), and hormonal fluctuations (Yesilbas & Oguz 2022). However, most of these studies have focused on the physicochemical properties of the aquatic environment, with limited investigation into stress factors and stress physiology.

Iron is an essential trace element in fish, playing a critical role in numerous physiological and biochemical processes. It is a key component of haemoglobin and myoglobin, which are responsible for oxygen transport and storage, respectively. Fish obtain iron primarily through the diet, although waterborne uptake via the gills may also contribute under certain environmental conditions. Adequate iron availability is therefore vital for aerobic metabolism, swimming performance, and overall growth. Iron also functions as a cofactor for many enzymes involved in mitochondrial respiration, DNA synthesis, and antioxidant defense. In fish, iron homeostasis is tightly regulated because both iron deficiency and excess can be detrimental. Iron is particularly important in migratory fish because long-distance migrations require high aerobic capacity and sustained swimming performance, which depend on efficient oxygen transport and mitochondrial energy production mediated by iron-containing proteins such as haemoglobin and cytochromes (Bury & Grosell 2003; Serna-Duque et al. 2022; Wang et al. 2023).

Therefore, this study aimed to investigate metabolic changes of pearl mullets during their anadromous migration for spawning. Fish were captured just entering a freshwater stream (pre-spawning) and before returning to the lake (post-spawning). Thus, this study deals with osmoregulatory shift, response of the antioxidant defence, histological changes, and finally the metabolic cost of anadromous spawning migration of pearl mullets. One of the main objectives of this study is to contribute to the conservation efforts of the endemic and endangered pearl mullet (*A. tarichi*), emphasising its vulnerability.

## Materials and methods

### Experimental fish

**Fig. 1:**
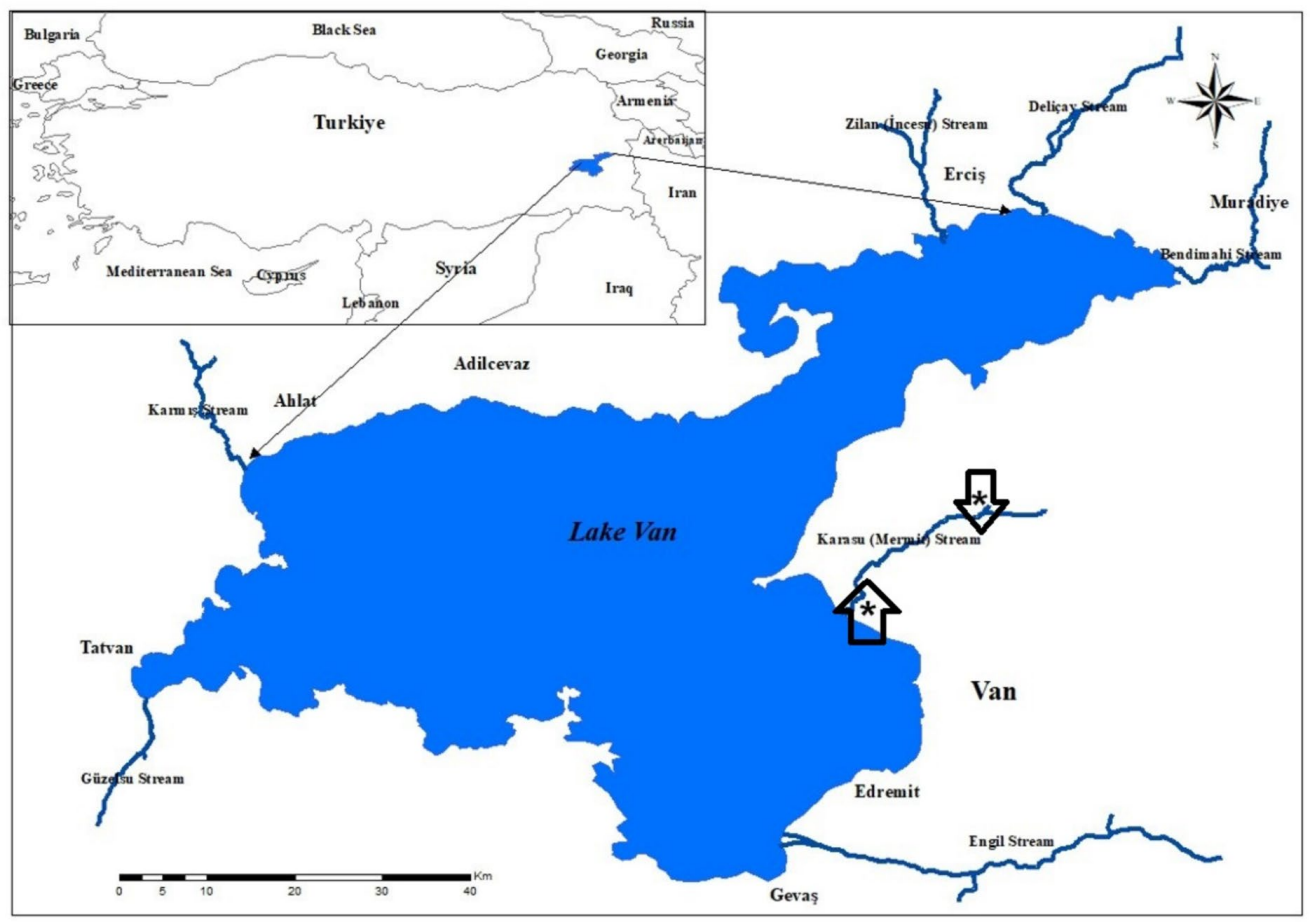
A map of Lake Van (3.775 km^2^) and sampling stations in the Karasu River of pearl mullets. Asterisk in the up arrow shows the pre-spawning sampling station (river mouth), while the asterisk in the down arrow shows the post-spawning station

Pearl mullet (*A. tarichi*) were captured from the Karasu Stream, which flows into Lake Van, during the reproductive period between April and July (2024). Fish were sampled during the pre-spawning period (weighed between 73 and 156 g and fork lengths between 15 and 22.5 cm) and the post-spawning period (weighed between 50 and 93 g and fork lengths between 16.5 and 21 cm). Fish were captured using cast nets near the freshwater inlets. After spawning, they were captured from the upper parts of the Karasu River using the same method (Fig. 1). As the fish is an endemic and endangered species, the necessary permissions for this study were obtained from the Ministry of Agriculture and Forestry and the Local Animal Ethics Committee of Van Yüzüncü Yıl University (Decision No. 2024/01–40 dated 01.02.2024).

The mean size and fork length of fish (5 male, 5 female) did not change significantly between the two spawning. The captured specimens were transported in oxygenated containers to the Biology Department, Faculty of Science, Van Yuzuncu Yil University. Anaesthesia of fish was done as described in our previous paper (Erdemir and Oguz, 2026). Then, fork length and total body weight were measured, and fish were dissected using sterile instruments. The gonadosomatic index (GSI) and the hepatosomatic index (HSI) values were calculated according to Aydin et al. (2023). Gill tissue (comprising all filaments on one side), muscle tissue (from the right dorsal region), liver, brain, kidney, and gonad tissues were dissected out and frozen at − 80 °C in an ultra-freezer (Esco UUS-480A) for subsequent analysis.

### Measurement of ATPase activity

Tissues were homogenised on ice in a homogenisation buffer containing 250 mM sucrose and 20 mM Tris base (pH 7.8), at a 1:10 (w/v) ratio, using a homogenizer set to 9500 rpm for 1.5 min. The homogenates were transferred to Eppendorf tubes and centrifuged at 13,000 × g for 20 min at + 4 °C. The resulting supernatants were used for ATPase activity and total protein content analysis. ATPase activity in tissues was determined based on the quantification of inorganic phosphate (Pi) released during ATP hydrolysis, using KH₂PO₄ as a standard (Atkinson et al. 1973). The amount of Pi was measured spectrophotometrically at a wavelength of 390 nm. The final concentrations of reagents in the incubation medium for Na⁺/K⁺-ATPase and Mg^2^⁺-ATPase activity measurements were as follows: 40 mM Tris–HCl (pH 7.7), 120 mM NaCl, 20 mM KCl, 3 mM MgCl₂, 3 mM ATP, and 1 mM ouabain. Incubations were performed in both the presence and absence of 1 mM ouabain (a specific inhibitor of Na⁺/K⁺-ATPase). Total ATPase activity was measured in the absence of ouabain, while Mg^2^⁺-ATPase activity was assessed in its presence. The difference between these two values was accepted as Na⁺/K⁺-ATPase activity. Ca^2^⁺-ATPase activity was determined following a similar principle, with modifications to the incubation medium (Pepe et al. 2022). Specifically, the difference between activities measured in the presence of 1 mM EGTA and 1 mM CaCl_2_ versus those without CaCl_2_ was considered Ca^2^⁺-ATPase activity. Total protein concentrations in all tissues were determined using the method of Lowry et al. (1951).

### Measurement of AChE activity

AChE activity in muscle and brain tissues was measured using the method described by Ellman et al. (1961). Cholinesterase enzymes catalyse the hydrolysis of acetylthiocholine into thiocholine and acetate. The resulting thiocholine reacts with DTNB to produce 5-thio-2-nitrobenzoic acid, a yellow-coloured compound whose absorbance was measured at 412 nm over 1 min. AChE activity in fish tissues was measured as described by Yilmaz et al. (2015).

### Determination of total energy reserves

Total energy reserves in liver and muscle tissues (left dorsal region) were calculated as previously described (Emre et al. 2013). This approach involves determining the tissue concentrations of total protein, glucose, and lipid content, followed by conversion of these biomolecules into energy equivalents (Gnaiger 1983). Glucose levels were quantified using the Anthrone method (Plummer 1971), lipid content by the method of Van Handel (1985), and protein levels using the Lowry method (Lowry et al. 1951).

### Measurements of antioxidant enzyme activity

First, liver tissues stored at − 85 °C were thawed and homogenised in an ice bath using a buffer containing 100 mM KCl and 100 mM KH₂PO₄ (pH 7.4) at a ratio of 1/10 (w/v). This process was done at 9500 rpm for 5 min using a homogeniser (Ultra Turrax T-25). The homogenates were then centrifuged at 10,000 rpm for 30 min (Hettich Universal 30 RF), and the supernatants were carefully decanted to determine antioxidant enzyme activities. SOD activity was measured according to the method based on the inhibition of cytochrome C reduction at 550 nm for 1 min in a reaction medium containing 1 mL of 50 mM phosphate buffer (pH 7.8), 0.1 mM EDTA, 10 mM cytochrome c, 0.05 mM hypoxanthine, 1.87 mU mL^−1^ xanthine oxidase, and supernatant (McCord and Fridovich 1969). GPX activity was measured by monitoring the decrease in NADPH absorbance at 340 nm (*ε* = 6.22 µmol^−1^ cm^2^) in a final volume of 1 L containing 100 mM phosphate buffer (pH 7.4), 2 mM GSH, 0.12 mM NADPH, 2 U GR, supernatant, and 3 mM CHP (Livingstone et al. 1992). CAT activity was measured by recording the decrease in absorbance at 240 nm (*ε* = 0.0392 µmol^−1^ cm^2^) for 1 min in a final volume of 1 mL containing 75 mM phosphate buffer (pH 7.4), 25 mM H₂O₂, and 20 µL supernatant (Lartillot et al. 1988). GST activity was measured by monitoring the increase in absorbance at 340 nm for 1 min due to the conjugation of GSH and CDNB in a reaction medium containing 1 mL of 100 mM phosphate buffer (pH 7.4), 1 mM GSH, 1 mM CDNB, and supernatant (Habig et al. 1974). GR activity was also measured based on the same principle in a reaction medium containing 100 mM phosphate buffer (pH 7.4), 0.1 mM NADPH, supernatant, and 1 mM GSSG (Carlberg & Mannervik 1975). Protein levels were determined using the method of Lowry et al. (1951).

### Immunohistochemistry (IHC)

For IHC studies, PFA-fixed gill, liver, and intestine tissue were embedded in paraffin. Sections (5 µm) were made using the microtome and mounted onto poly-L-lysine-coated slides. For immunohistochemistry studies, Histostain-Plus Bulk Kit (85–8943, Invitrogen, USA) was used according to the kit instructions. Sections were washed in phosphate-buffered saline (PBS) and then incubated with 0.3% hydrogen peroxide in distilled water (DiH_2_O) for 20 min to inhibit endogenous peroxidase activity. After washing in PBS, the sections were incubated with kit-blocking serum at RT for 1 h. Sections were incubated overnight at RT with the monoclonal mouse antibody raised against the HSP70 (1:100; MA1-80,199, Thermo Scientific, USA). After washing in PBS, sections were incubated with kit biotinylated secondary antibodies and Streptavidin HRP conjugates for 10 min, rinsed with PBS, and incubated in 3,3ʹ-diaminobenzidine (DAB + Substrate Chromogen, K3468, Dako, Denmark) for 3 min. All sections were dehydrated, cleared, and coverslip-mounted with permanent mounting media. Sections were examined microscopically and photographed as described previously. Negative reagent control sections were incubated under the same conditions without a primary antibody.

### Histological examination

The fish were anaesthetised with phenoxy-ethanol (1:20,000, v/v) and sacrificed as described in our previous paper (Erdemir and Oguz, 2026). The gill, liver, and intestine tissues were quickly excised and placed in Bouin’s solution and 4% paraformaldehyde, then stored in 70% alcohol at + 4 °C until paraffin embedding. Tissues were passed through a graded alcohol series (70%, 80%, 90%, and 100%) and xylol, then embedded in paraffin blocks. Afterwards, 5-µm sections were cut from the paraffin blocks using a microtome (HM 325 manual microtome, microm International GmbH, Waldorf, Germany). The kidney sections were stained with hematoxylin and eosin and periodic acid-Schiff to assess the general histological structure and glycogens (Bancroft & Gamble 2002). The stained slides were covered with Entellan, examined under a light microscope (Leica DMI 6000B, Germany), and photographed with a Leica DFC 490 digital camera (Leica Microsystems, Germany).

### Statistical analysis

**Fig. 2:**
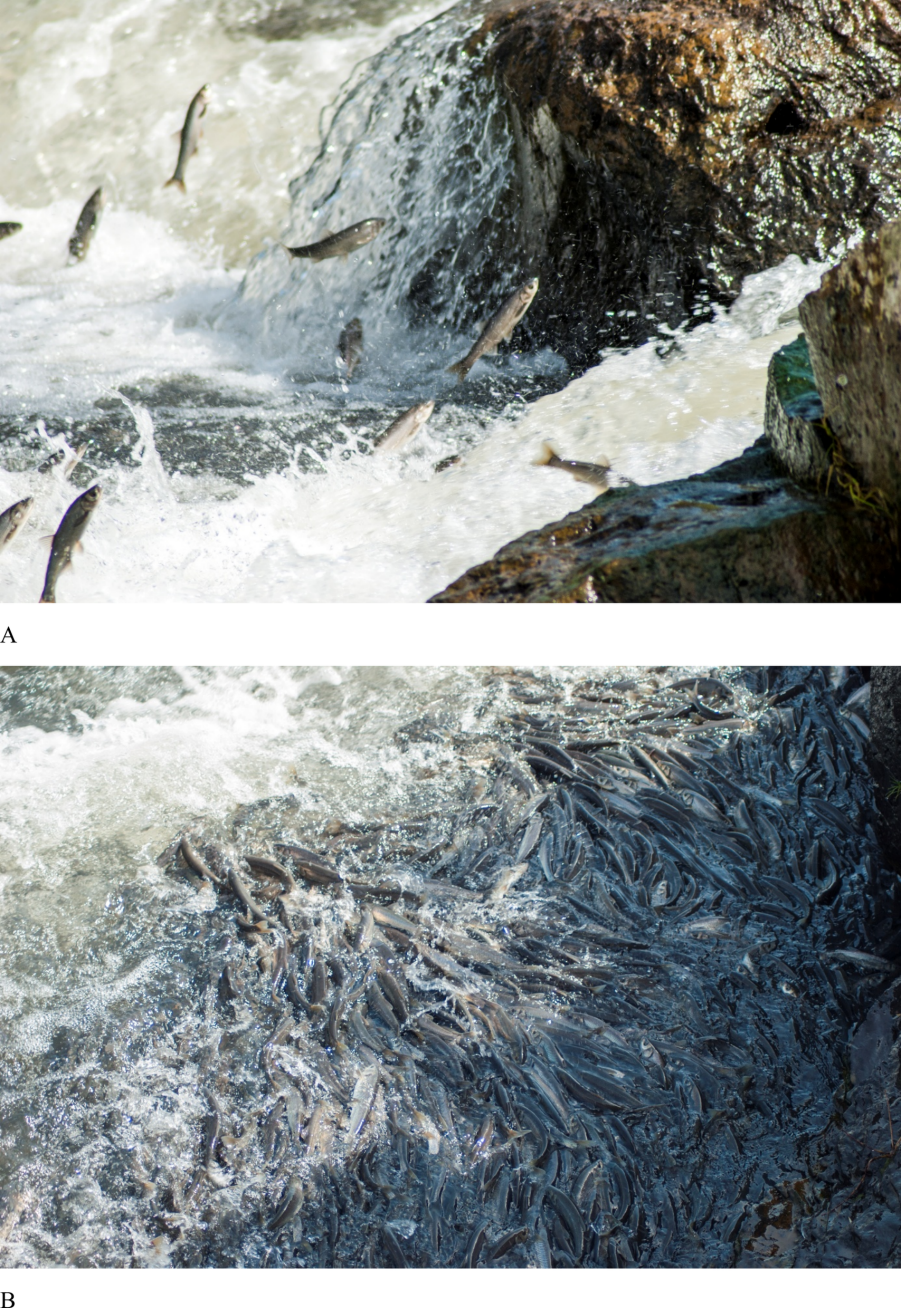
Pearl mullets (*Alburnus tarichi*) exhibit a remarkable behaviour by leaping upstream against the current (**A**) and swimming in flocks for spawning migration (**B**)

All statistical analyses were performed using SPSS version 20. Before statistical analysis, the distribution of the data was evaluated. The Mann–Whitney *U* test or Student’s *t*-test was applied to data depending on their distribution. All data were expressed as mean ± standard error, and statistically significant (*p* < 0.05) differences were highlighted in the figures (Fig. 2).

## Results and discussion

A total of 20 fish were captured in pre-spawning (5 ♀ and 5 ♂) and post-spawning (5 ♀ and 5 ♂) for each sampling. Fork lengths of pre-spawning fish ranged from 15 to 22.5 cm, while those of post-spawning fish ranged from 16.5 to 21 cm. Total body weights varied between 73–156 g in pre-spawning individuals and 50–93 g in post-spawning individuals. All sampled fish were older than 3 years. Although the size of fish between pre-spawning and post-spawning was very close in the two group samplings, the weights of fish decreased in post-spawning samples, suggesting weight loss due to releasing eggs and sperm. Table 1 shows the GSI and HSI values indicating significant differences between pre-spawning and post-spawning. This table demonstrates that GSI values were significantly (*p* < 0.05) lower in the post-spawning group. This suggests that fish successfully released their eggs and sperm into the water. Nevertheless, HSI values showed no significant (*p* > 0.05) differences between pre- and post-spawning groups. This may suggest that both sexes consumed similar amounts of reserves from the liver. As a result of histological examinations, no abnormalities in oocyte degeneration, perinucleolus, early cortical alveolus stages, and ovaries filled with somatic stromal tissue were detected. The absence of histological abnormalities in fish highlights that they were healthy individuals. At the same time, the differences observed between pre-spawning and post-spawning may emphasise that those might relate to reproduction physiology.

**Table 1:** GSI and HSI values in male and female pearl mullets. Asterisk indicates significant (*p* < 0.05) difference between pre-spawning and post-spawning in the same sex

Index	Male		Female	
	Pre-spawning	Post-spawning	Pre-spawning	Post-spawning
GSI	10.09 ± 0.35	3.26 ± 0.48*	21.54 ± 2.79	8.04 ± 0.47*
HSI	1.46 ± 0.12	1.52 ± 0.11	1.25 ± 0.083	1.41 ± 0.084

### Response of the osmoregulation system

**Fig. 3:**
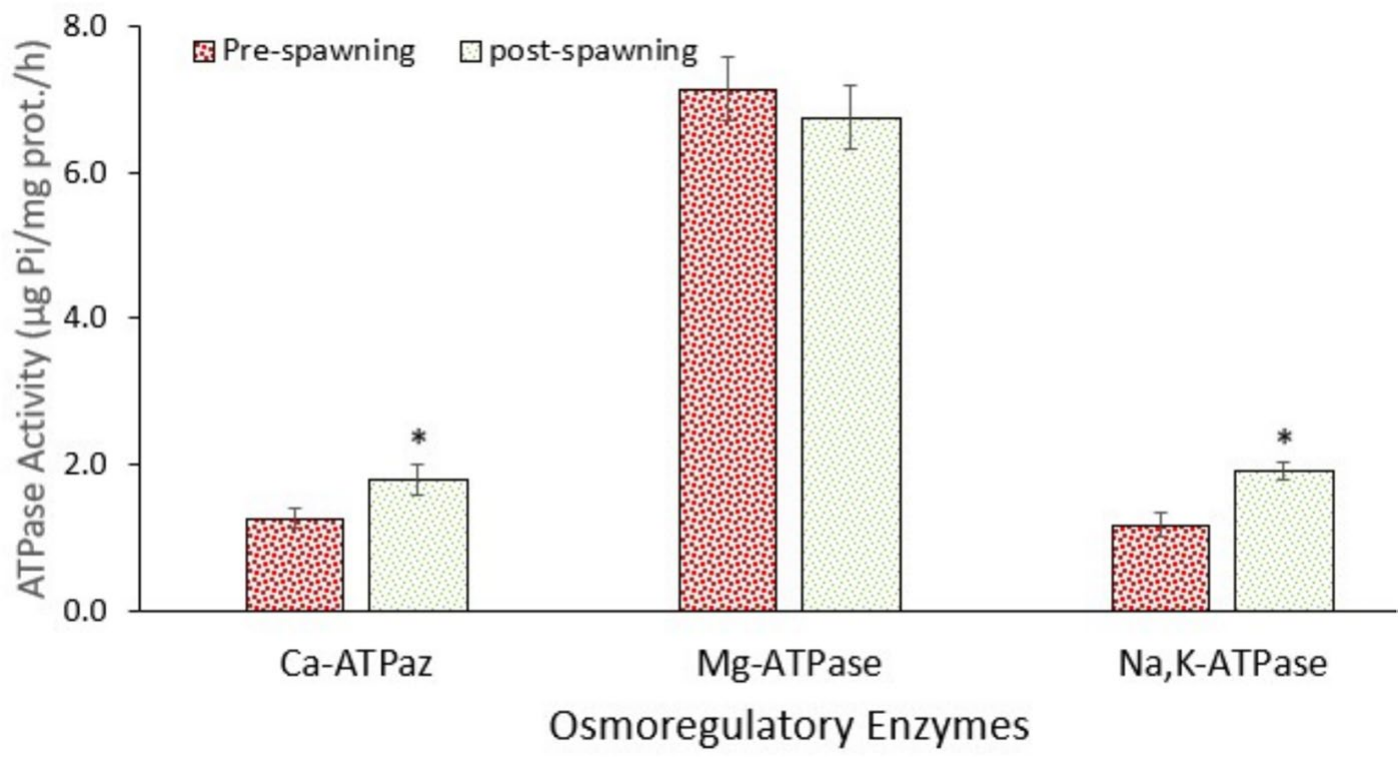
Activity of osmoregulatory enzymes in the gill of pearl mullets during spawning migration. Data are the mean and associated standard errors of 10 samples (5 male and 5 female). Asterisk indicates significant difference (*p* < 0.05) between pre-spawning and post-spawning fish

**Fig. 4:**
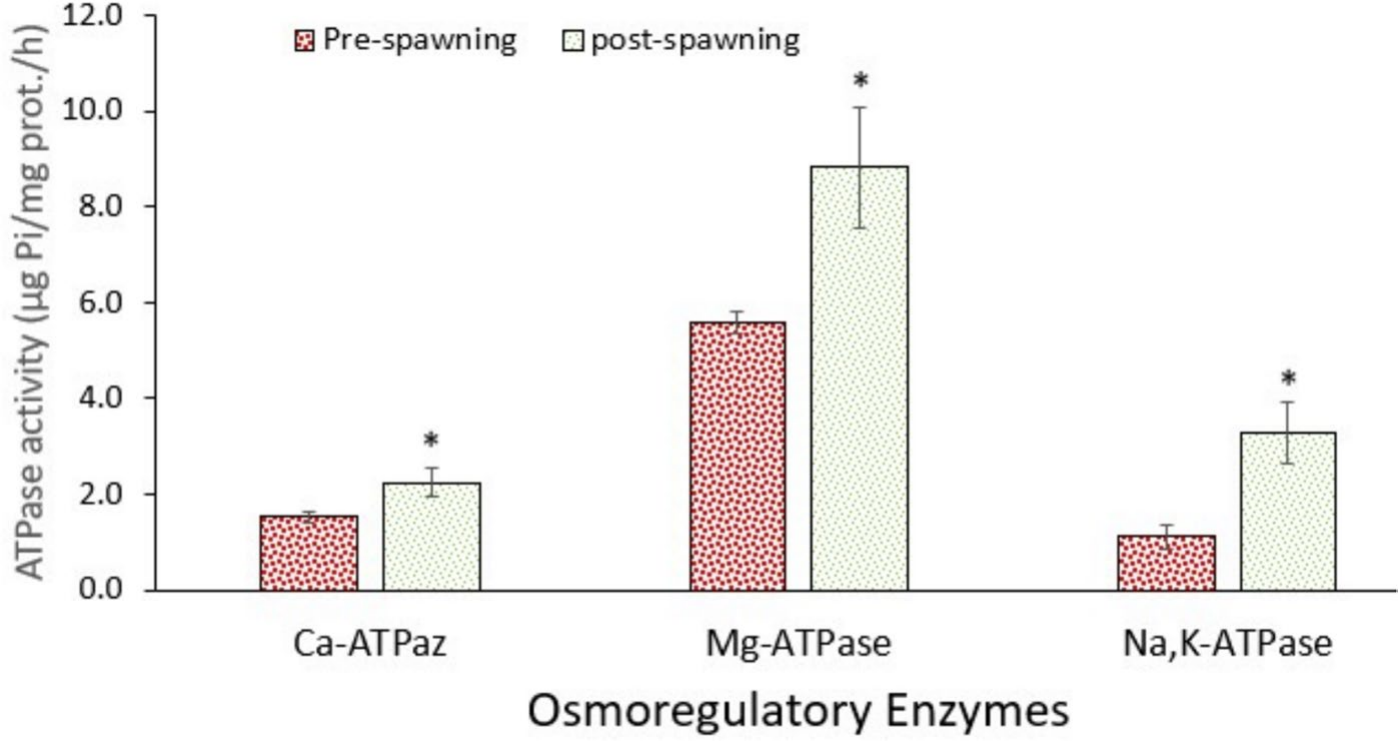
Activity of osmoregulatory enzymes in the kidney of pearl mullets during spawning migration. See Fig. 3 for details

**Fig. 5:**
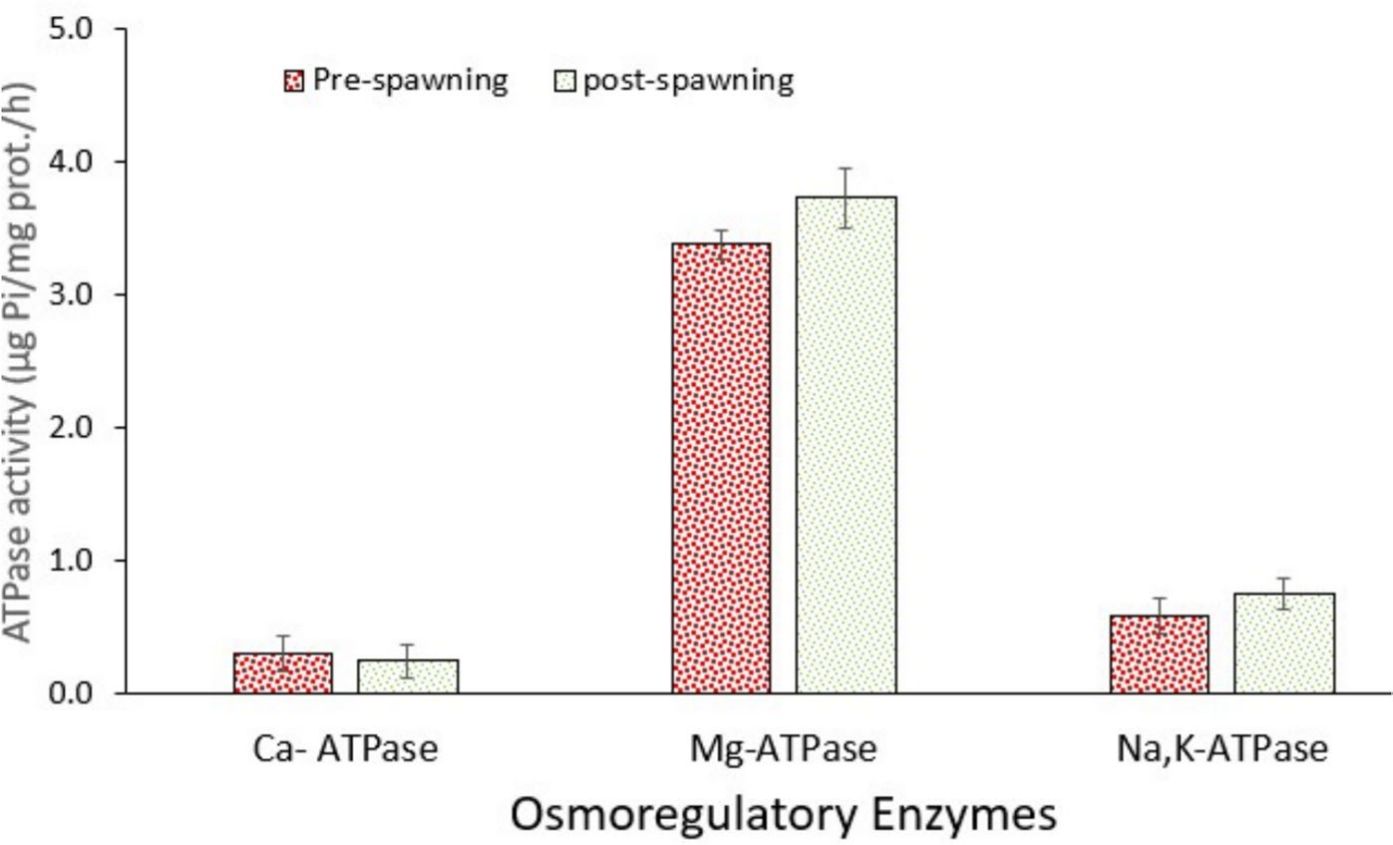
Activity of osmoregulatory enzymes in the brain of pearl mullets during spawning migration. See Fig. 3 for details

Ion-ATPases are very important enzymes in the ion balance between the interior and extracellular fluid of animals. In this context, the most important enzymes are Na⁺/K⁺-ATPase, Mg-ATPase, and Ca-ATPase. Data showed that the activities of ATPases changed between pre-spawning and post-spawning groups, except for the brain (Figs. 3, 4, and 5). Figure 3 shows that the activities of Na⁺/K⁺-ATPase and Ca-ATPase in the gill increased significantly (*p* < 0.05) in post-spawning fish, whereas Mg-ATPase activity did not change (*p* > 0.05). However, all ATPase activities increased significantly (*p* < 0.05) in the kidney in post-spawning fish (Fig. 4). Nevertheless, no significant change occurred in the brain (Fig. 5). It is well known that migratory fish undergo hormonally regulated physiological changes during migration that adjust their osmoregulatory systems for transition from freshwater to seawater or brackish water, though the level of physiological alterations may change depending on species (Hourdry 1995). Brönmark et al. (2014) also emphasised that the migration of fish causes high levels of metabolic adaptations after following changes in habitat. In this context, Yesilbas and Oguz (2021) pointed out the alterations in the levels of hormones affecting the osmoregulation of Pearl mullet during the spawning period. Additionally, further changes in gonadal development and histological changes were also demonstrated (Unal et al. 2007; Çelebi & Oğuz 2020; Alkan & Oğuz 2021). Similarly, Christensen et al. (2019) indicated that salinity changes reduced swimming performance in low salinity–tolerant European perch but not in high salinity–tolerant ones, with no significant metabolic cost detected. These intra-specific differences highlight the importance of considering population-specific traits in the conservation of estuarine fish. As in the case of Pearl mullet, several other factors influence fish metabolism. Oğuz and Kaval Oguz (2020) indicated that before spawning, Pearl mullet are exposed to various stress factors, including high population density, predators, microbial and parasitic infections, and intense energy expenditure due to swimming against the current.

Every spring, Pearl mullet migrate to rivers that flow to Lake Van for spawning. After spawning, they return to Lake Van (high alkalinity and salinity), which demands active osmoregulation to cope with changing ion levels. At this point, ATPases are upregulated to excrete or absorb excess salts and re-establish osmotic balance across epithelial tissues. After spawning, fish often experience osmotic stress due to energy depletion, hormonal changes, and changes in water/ion exchange needs (Hourdry 1995; Brönmark et al. 2014). As occurred in the present study, increased ATPase activity helps restore ionic homeostasis and recover from reproductive exhaustion. Additionally, post-spawning may involve gill remodelling and increased mitochondria-rich cells, which are sites of active ion transport. This correlates with elevated ATPase activity, supporting enhanced ion exchange.

### Antioxidant system response

**Fig. 6:**
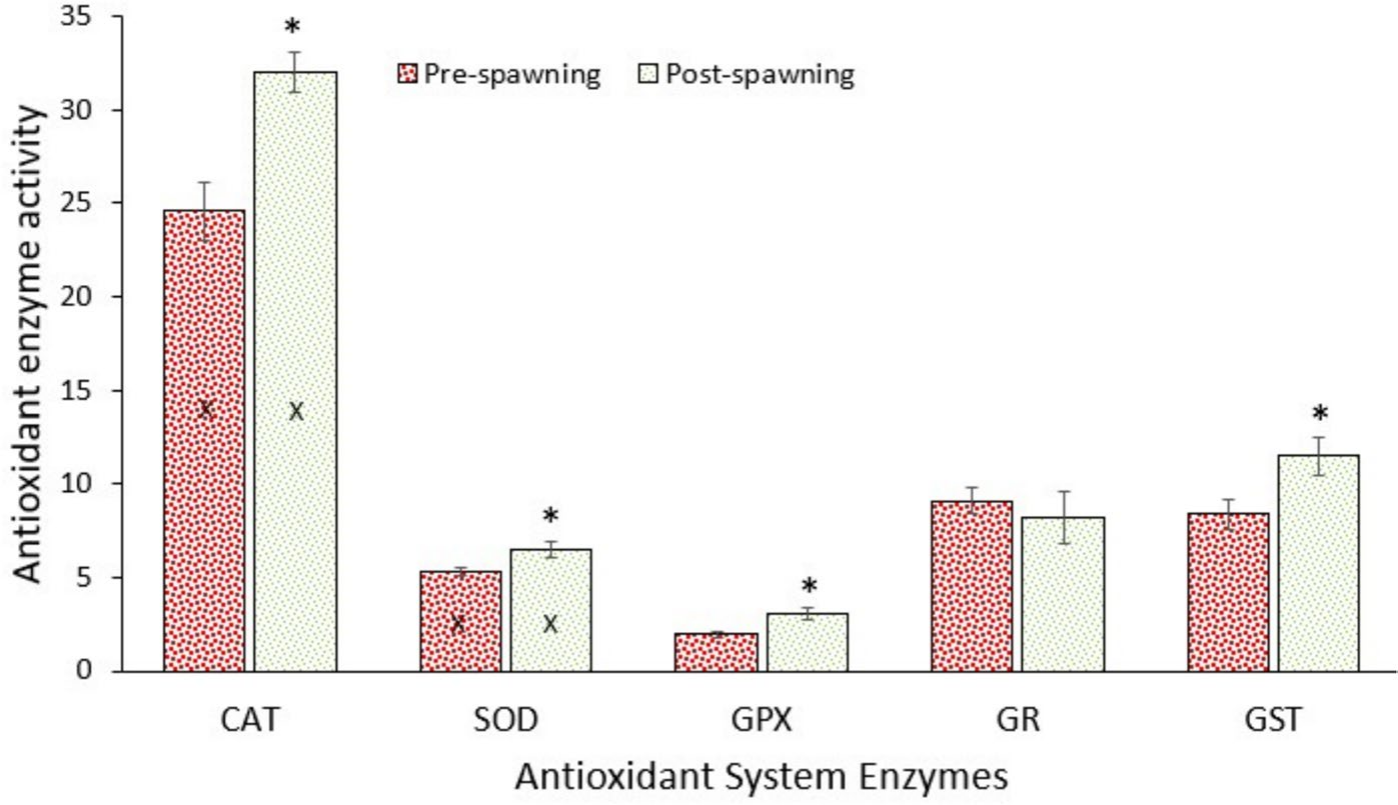
Activities of CAT (µmol H_2_O_2_/g w.w./min), SOD (U/g w.w.), GPX (µmol/g w.w./min), GR (µmol/g w.w./min), and GST (µmol/g w.w./min) in the liver of pearl mullets during spawning migration. Values containing X should be multiplied (CAT by 1000 and SOD by 100). See Fig. 3 for details

Data showed that the antioxidant system enzymes in the liver of fish significantly increased (*p* < 0.05) after spawning, except GR activity (Fig. 6). The activity of CAT in the liver of pearl mullet was considerably higher when compared to other fish species in both spawning samples. Similarly, SOD activity was also high in this species. As it is known, CAT and SOD are the first defence line in the antioxidant system response to eliminate oxidants that may occur during high respiration (Saglam et al., 2014). The significant post-spawning increase in antioxidant enzyme activities reflects a typical oxidative stress response linked to the physiological burden of reproduction, migratory effort, and environmental transitions. Reproduction, particularly in migratory or energetically demanding species like the Pearl mullet, causes extensive metabolic upregulation, leading to an increase in reactive oxygen species (ROS) production. ROS are byproducts of aerobic metabolism and, if unregulated, can cause oxidative damage to lipids, proteins, and DNA. Data suggest that the fish are actively compensating for increased oxidative stress following spawning. The reproductive period, especially migration and gamete release, elevates oxygen consumption and mitochondrial respiration, leading to excess ROS. The antioxidant defense system is upregulated to counteract this. After spawning, fish undergo tissue remodelling and immune system activation, both of which generate oxidative byproducts. Increased antioxidant enzyme activity supports cell protection and recovery. As Pearl mullet migrate between different salinity zones, these environmental stressors may exacerbate oxidative stress, triggering antioxidant responses. In a previous study, Oguz et al. (2019) demonstrated the total oxidant status of pearl mullet in pre-spawning samples, while the total antioxidant status was higher in post-spawning samples. Similarly, Kaptaner and Dogan (2019) found that pearl mullet had oxidative stress during spawning migration as the levels of MDA and antioxidant enzymes increased after spawning. Birnie-Gauvin et al. (2017) examined the link between oxidative stress and migration in semi-anadromous brown trout, a species where some individuals migrate to sea while others remain in streams. They found that fish that later migrated had higher antioxidant capacity than non-migrants, and earlier migration was associated with higher antioxidant capacity and smaller body size. These results suggest that oxidative status influences both migration strategy and timing well before migration begins. Kaptaner et al. (2022) pointed out that antioxidant mechanisms of pearl mullets play significant roles during the acclimation of the gland to different environments, such as Lake Van to freshwater.

### Iron levels

**Fig. 7:**
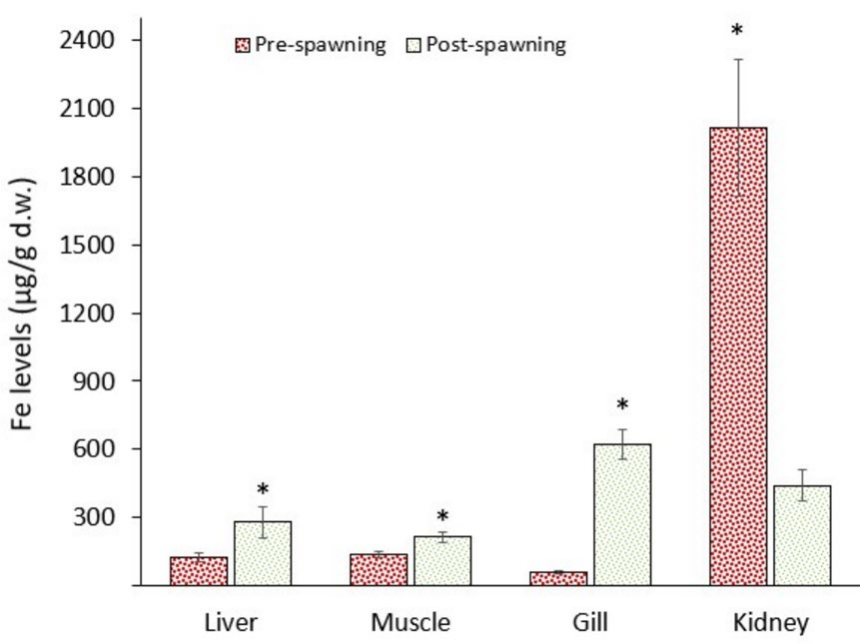
Fe levels in the tissues of pearl mullets during spawning migration. See Fig. 3 for details

Iron is an essential metal for fish metabolism, as it is a part of haemoglobin, a molecule responsible for oxygen transport. Oxygen is directly related to cellular respiration in aerobic metabolism. This means that Fe and oxygen are involved in energy (ATP) production, which is then used for metabolic activities. The results of this study showed that Fe levels in the kidney were significantly (*p* < 0.05) higher in the pre-spawning samples compared to the post-spawning ones. On the other hand, Fe levels in the gill, muscle, and liver were significantly (*p* < 0.05) higher in post-spawning samples (Fig. 7). Data suggest that there was a relationship between Fe and energy levels, as the Fe levels increase (oxygen carrying capacity), ATP synthesis also increases through cellular respiration. It could be evaluated that high Fe levels (haemoglobin) in the kidney were due to high activity of the kidney while fish migrate to the river, which requires extra energy to compensate for the osmoregulation shift. However, data also show that Fe levels increased in the other tissues in post-spawning fish, emphasising the energy requirements of all tissues. As it is well known, iron’s primary function as a core component of haemoglobin links it directly to oxygen transport, and therefore to aerobic ATP production. This makes Fe levels a valuable proxy for oxygen demand and energy metabolism in tissues (Lall & Kaushik 2021). The elevated Fe concentration in the kidney of pre-spawning fish likely correlates with intensified osmoregulatory activity during upstream migration. Kidney function is critical in maintaining internal ionic and water balance as fish transition between saline and freshwater environments. This process demands high energy input, which would explain the higher oxygen and Fe demand during this period. Following spawning, the observed increase in Fe levels in the gills, muscle, and liver suggests a shift in metabolic demand from migratory preparation to recovery and tissue maintenance. For example, the gills require Fe to support ion exchange and gas exchange, particularly under post-spawning stress and osmoregulatory imbalance. Muscles recover from prolonged migration and spawning-related activity and likely demand more oxygen for repair and ATP generation (Lall & Kaushik 2021). The liver is central to metabolic detoxification, glycogen mobilisation, and protein turnover processes that are enhanced post-spawning as the fish attempts to restore homeostasis. In summary, Fe is tightly linked to haemoglobin function and oxygen delivery, and changes in its distribution likely mirror oxygen-dependent ATP production in different organs. The study of Wilson et al. (2014) supports the present findings on Fe metabolism. They pointed out that during long spawning migrations, fish mobilise body reserves and show signs of oxidative stress. Iron can be redistributed between blood, spleen, liver, and muscle and is associated with increased ferritin and oxidative damage. Standal et al. (1997) also agree that various stress factors may cause increased tissue iron levels, ferritin upregulation, haemolysis, and reduced haemoglobin changes that are linked to oxidative stress and impaired physiology. Teien et al. (2008) emphasised that iron speciation affects tissue Fe accumulation and toxicity, so both physiological mobilisation and external iron sources can change whole-body Fe during stress or migration.

### Nerve system response

**Fig. 8:**
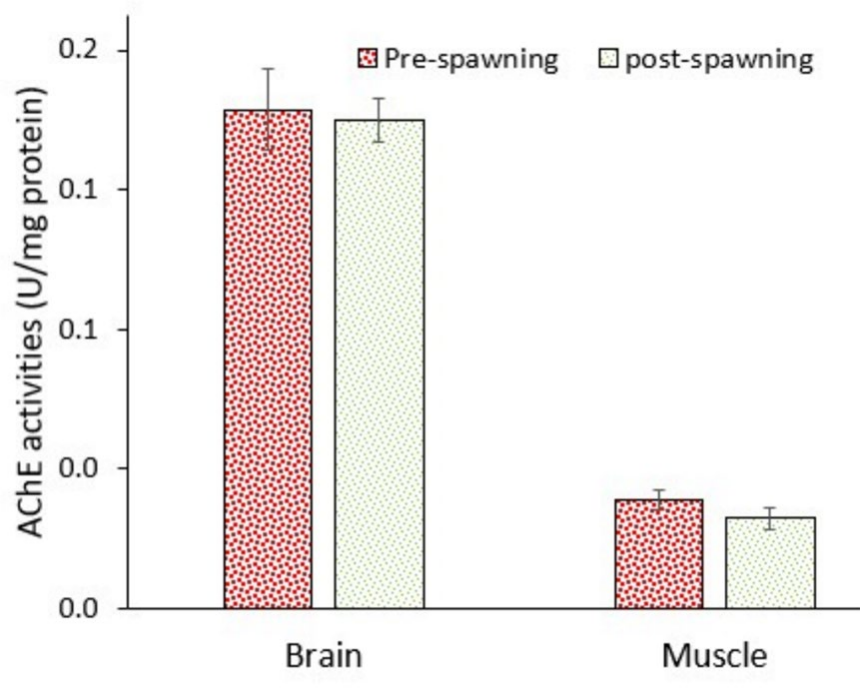
Activity of neurotransmitter enzymes in the brain and muscle of pearl mullets during spawning migration. See Fig. 3 for details

Data showed that AChE activities in the brain and muscle did not change significantly between pre- and post-spawning fish (Fig. 8). AChE activity is very important for muscle movement and impulse signalling through nerves. It needs large amounts of ATP energy to do this task. It seems that fish metabolism tried to keep AChE activity constant, which is necessary for muscle contraction and impulse signalling. Data revealed that AChE activity remains stable in both brain and muscle tissues despite significant metabolic changes, emphasising the importance of vital neural and muscular functions. As it is known, AChE is essential for neurotransmission and muscle control. Unlike other systems (e.g. energy storage or osmoregulation), which can tolerate temporary suppression or fluctuation, neural communication must be maintained for survival. Even minimal swimming or predator avoidance requires functional nerve-muscle signalling. Post-spawning fish may enter a catabolic state, conserving energy by reducing non-essential processes (e.g. growth, reproduction). However, metabolic resources are still directed toward maintaining AChE function, even when glucose and lipid levels decline. This reflects selective metabolic investment in systems crucial for immediate survival. Constant AChE activity may also indicate that neuronal structures and synapses remain functionally intact during the spawning period, though tissues like the liver and muscle undergo energy depletion in post-spawning fish, emphasising the critical biological roles of AChE. Formicki et al. (2025) reviewed the neurotoxicity of various pollutants in fish, emphasising the sensitivity of AChE under oxidative stress. Nevertheless, there is no evidence on the behaviour of AChE during migration in freshwater. Nevertheless, Östholm (1992) noted changes in AChE-positive cells across developmental/migratory stages (presmolt vs. postsmolt) in the pineal organ of Pacific coho salmon. Likewise, Krasnov et al. (2024) reviewed the smoltification physiology in Atlantic salmon, emphasising the changes in the neuroendocrine and immune system linked to downstream migration of salmon. Although AChE is highly sensitive to pollutant exposures or various stress factors (Tierney & Pyle 2024; Rao et al. 2005), no alteration in its activity in the brain and muscle of pearl mullets suggests that muscle contraction is one of the most important factors during migration, and loss in enzyme activity can be compensated for by increasing the turnover rate.

### Alteration in energy reserves

**Fig. 9:**
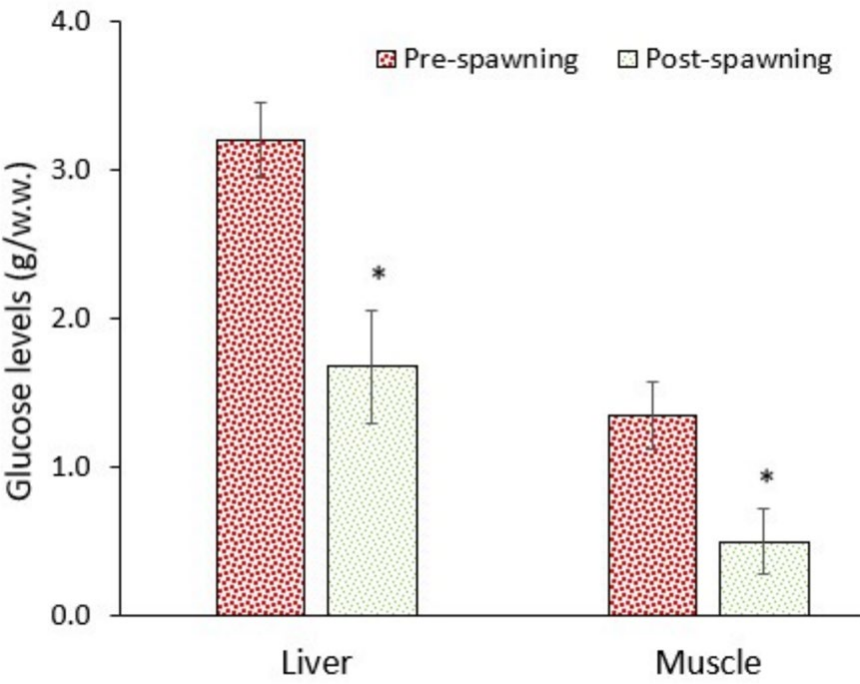
Glucose levels in the liver and muscle of pearl mullets during spawning migration. See Fig. 3 for details

**Fig. 10:**
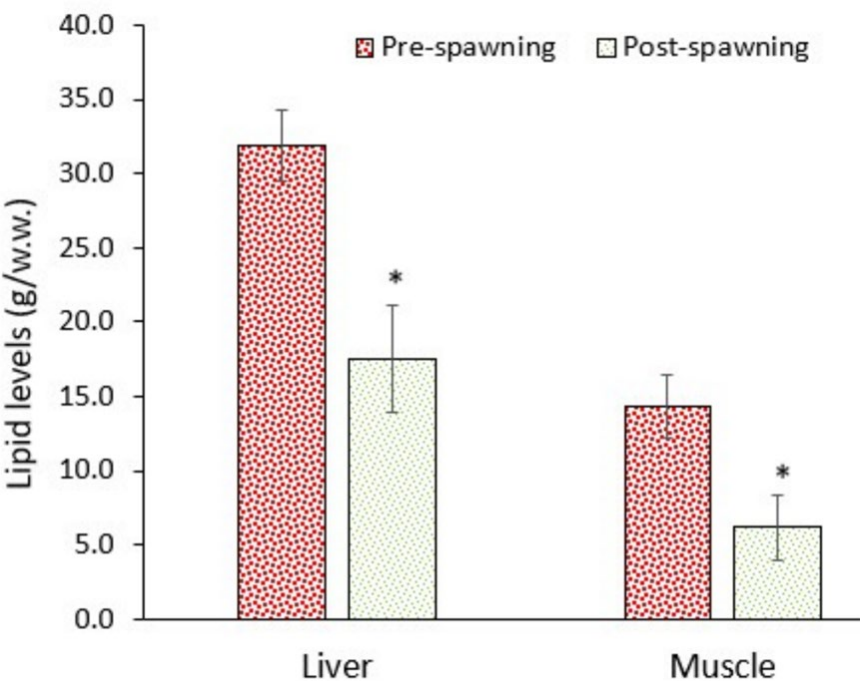
Lipid levels in the liver and muscle of pearl mullets during spawning migration. See Fig. 3 for details

**Fig. 11:**
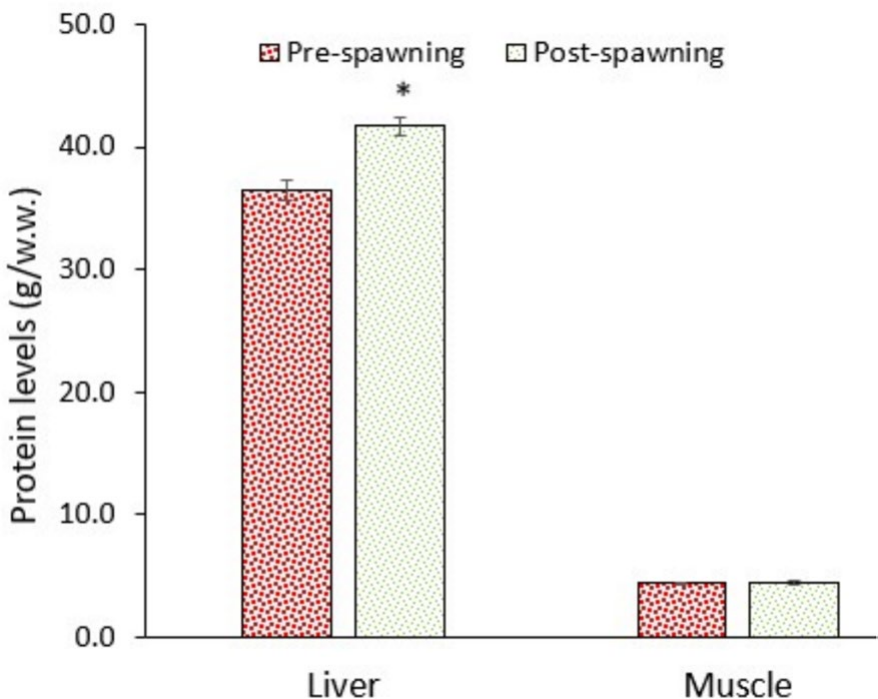
Protein levels in the liver and muscle of pearl mullets during spawning migration. See Fig. 3 for details

**Fig. 12:**
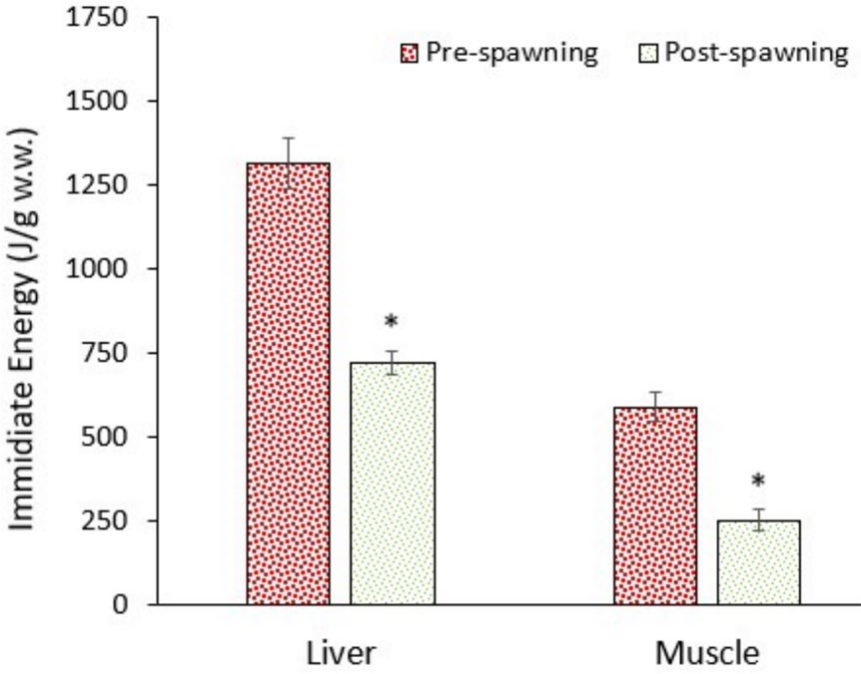
Immediate energy (derived from glucose and lipid) levels in the liver and muscle of pearl mullets during spawning migration. See Fig. 3 for details

Migration between a saline-alkaline water (Lake Van) and a freshwater river requires too much energy to cope with the shift in osmoregulation and other metabolic alterations. The data of this study supported this assumption. The levels of glucose and lipids in the liver and muscle significantly (*p* < 0.05) decreased in post-spawning fish, though protein levels in the liver increased (Figs. 9, 10, and 11). Consequently, the immediate energy reserves (derived from glucose and lipids) of the liver and muscle significantly (*p* < 0.05) decreased (Fig. 12). Data suggest that fish utilised substantial amounts of glucose and lipids to supply enough energy (ATP) to the metabolism. As it is well known, animals consume substantial amounts of energy under stressful conditions (Bossuyt and Janssen 2004; Canli et al. 2023).

Euryhaline species (like Pearl mullet) are capable of surviving in a wide salinity range thanks to flexible and dynamic ATPase systems. However, osmoregulation is energetically costly, and increased ATPase activity post-spawning reflects both the energy demand for ion regulation and the need to repair/maintain homeostasis after reproductive effort. This means that reproduction is an energetically expensive process, as migration, gonad development, mating behaviours, and sometimes nest building or territorial defence need extra energy. Thus, the decrease in immediate (glucose and lipid) energy stores might be due to high metabolic activity during spawning and elevated glycolysis and oxidative phosphorylation to meet energy demands. Additionally, lipids, especially triglycerides, are mobilised from muscle and liver tissues for energy production and oocyte development, as female fish allocate large amounts of lipids to form vitellogenin and egg yolk, essential for embryo development. Hinch and Rand (1998) indicated that swim speed patterns significantly influenced migration efficiency in sockeye salmon (*Oncorhynchus nerka*), with higher energetic costs associated with males, smaller fish, earlier migration years, and river reaches containing flow-constricting features. Kiessling et al. (2024) found that during long-distance spawning migration of sockeye salmon (*Oncorhynchus nerka*) showed strategic energy use by progressive muscle fibre reduction, selective lipid mobilisation, and region-specific shifts in enzymatic capacity during challenging passages like the Fraser Canyon.

### Changes in histology and HSP70 cells

**Fig. 13:**
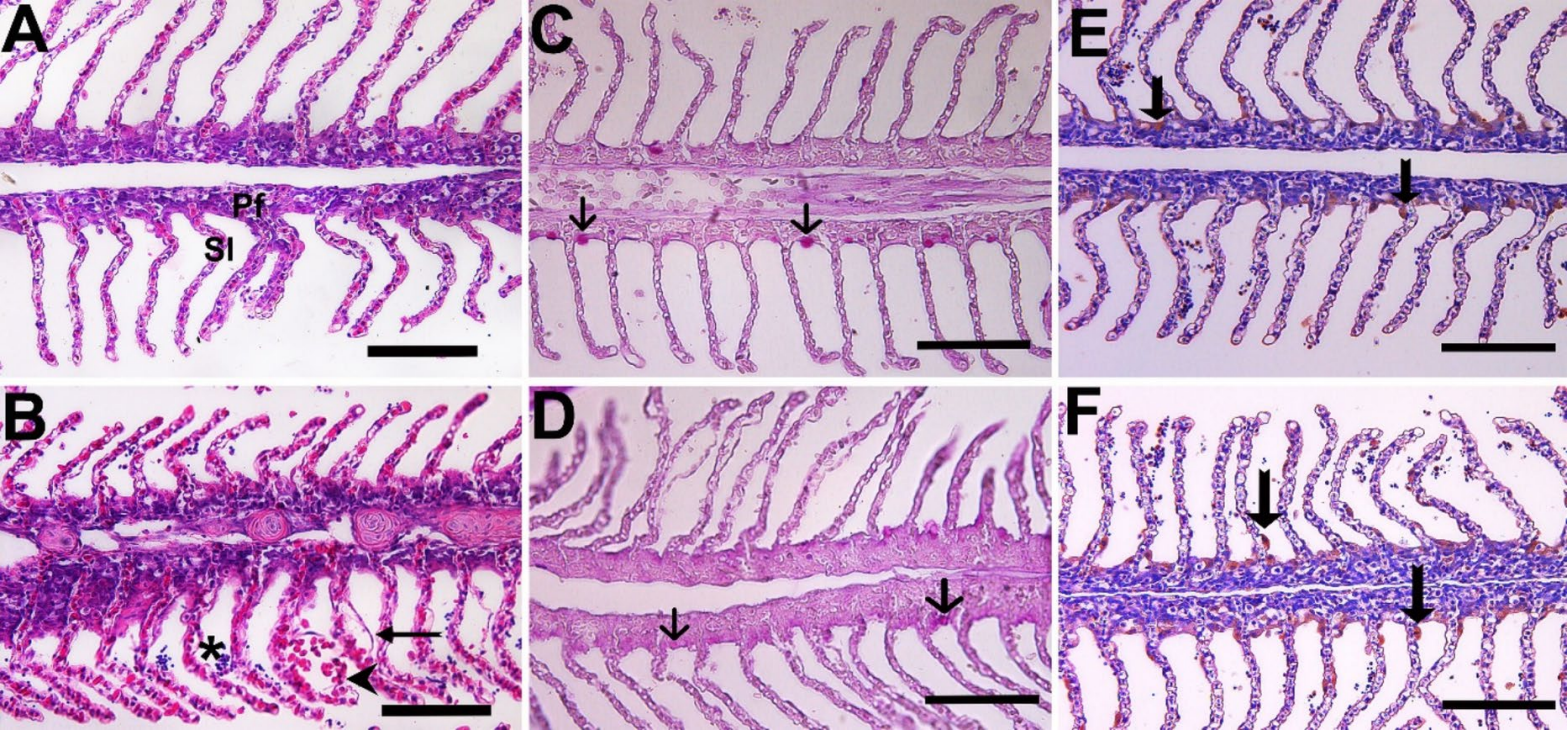
Histological examinations in the gill of pearl mullets during pre-spawning (**A**, **C**, **E**) and post-spawning (**B**, **D**, **F**). **A**, **B** Pf, primer filament; Sl seconder lamellae; *necrosis, arrow lamellar lifting, and arrowhead aneurysms (H&E). **C**, **D** Arrow mucous cell (PAS). **E**, **F** Arrow HSP70 + cells (immunohistochemistry). Bar: 50 µm

Histological examination of Pearl mullet tissues revealed some differences between the pre- and post-spawning groups. Lamellar separation and fusion in the gills were higher in the post-spawning groups (Fig. 13A and B). Lamellar separation is a reversible damage that reflects differences in the aquatic environment. Lamellar fusion is a protective mechanism observed after hypertrophy in gill cells. It is a mechanism developed by fish to protect tissue from environmental stress factors such as pollution, microbial, and parasitic infections observed in the aquatic environment (Oguz 2015; Sales et al. 2017). Gills are organs in direct contact with water and are directly affected by physicochemical changes in water, parasitic and bacterial environments, and applications. Glycoprotein-containing mucus secretions are secreted by the gill mucus. The mucus forms a protective layer by surrounding the gills as a protective barrier. In addition to this protection, it also functions in respiration, iono- and osmoregulation (Dang et al. 2020). Differences in mucus cell count and mucus density were observed in the Pearl mullet after spawning (Fig. 13C and D). This change is a response to various stress parameters.

**Fig. 14:**
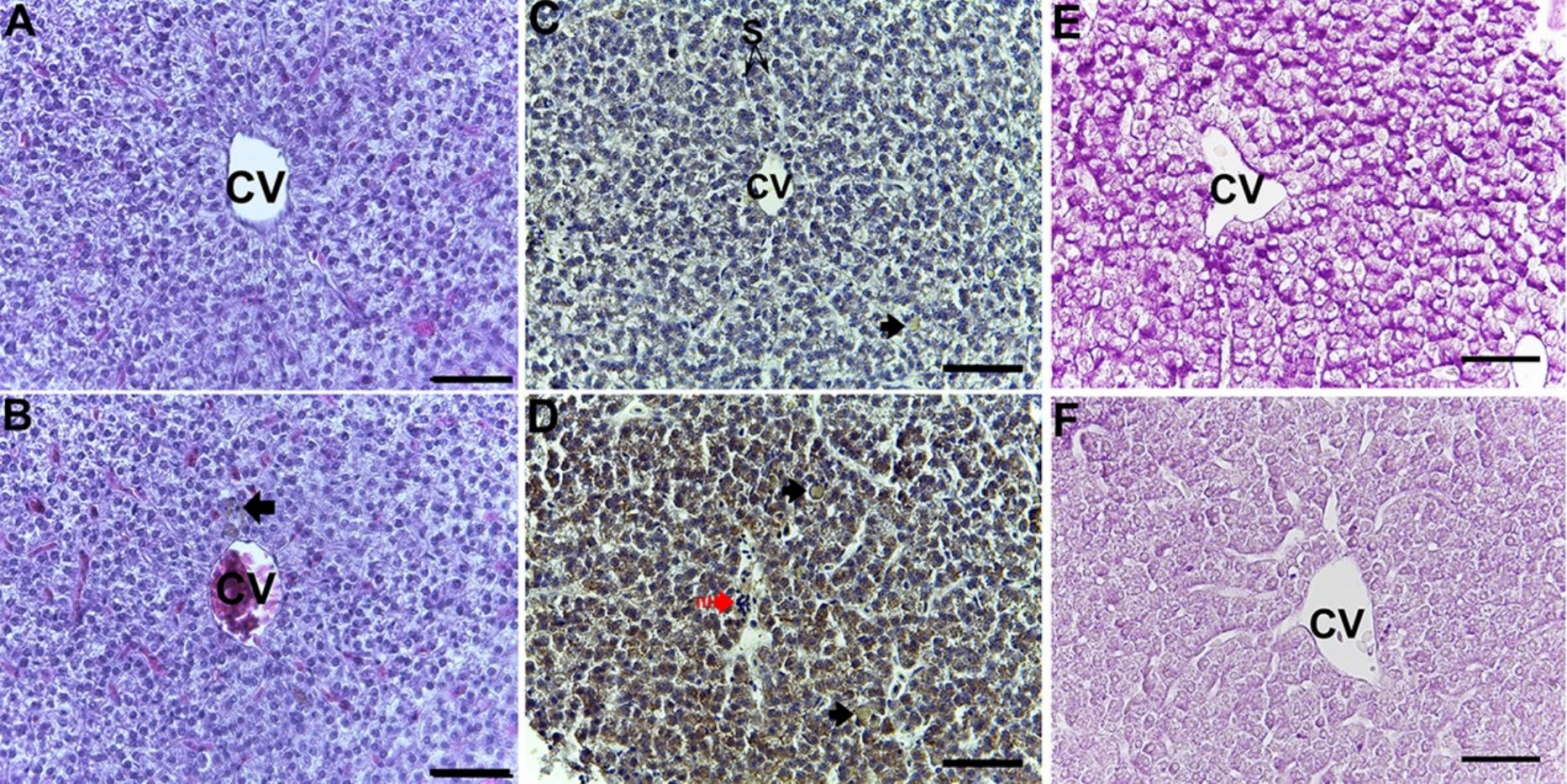
Histological examinations in the liver of pearl mullets during pre-spawning (**A**, **C**, **E**) and post-spawning (**B**, **D**, **F**). **A**, **B** H&E. **C**, **D** HSP70 immunohistochemistry. **E**, **F** PAS. CV, central vein; S, sinusoid; black arrow melanomacrophage centres, red arrow mononuclear cell infiltration. Bar 50 µm

**Fig. 15:**
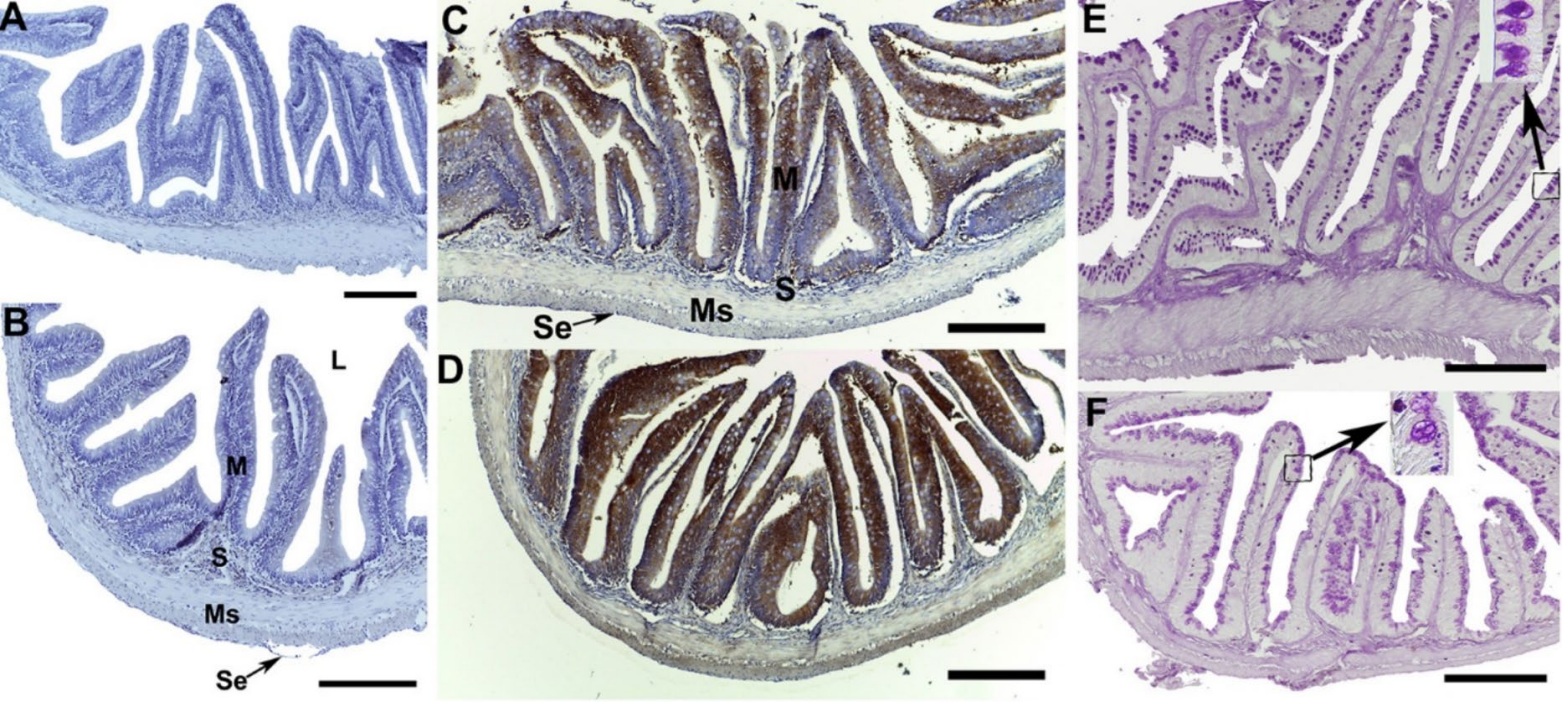
Histological examinations in the digestive tract of pearl mullets during pre-spawning (**A**, **C**, **E**) and post-spawning (**B**, **D**, **F**). **A**, **B** H&E. **C**, **D** HSP70 immunohistochemistry. **E**, **F** PAS. Se, serose membrane; Ms, muscularis layer; S, submucosa; M, mucosa; L, lumen. Bar: 50 µm

Mononuclear cell infiltration and melanomacrophage cell migration were observed in the liver tissue of fish, although small in size (Fig. 14A and B). These phenomena generally increase in size and frequency under environmental stress conditions and are considered reliable biomarkers of water quality (Agius and Roberts 2003). A decrease in glycogen content was also observed in hepatocytes (Fig. 4E, F). Spawning migration in anadromous fish is a highly complex and energy-consuming process. When swimming upstream, the Pearl mullet does not receive external food and faces predator pressure. This decrease in glycogen content may be an indicator of energy requirements in fish that do not receive external food during their spawning migration (Barcellos et al. 2010; Dhayalan et al. 2024). When the digestive tracts of Pearl mullet were examined in both groups, no histological differences were observed (Fig. 15A and B). However, a difference in the number and size of mucus cells was observed (Fig. 15E and F). Mucus secreted from mucus cells is known to have many functions. These include lubrication of the digestive tract, digestion, absorption, control of infectious diseases, and control of harmful or opportunistic microorganisms (Cao and Wang 2009). It can be said that different types of mucus cells observed in Pearl mullet play a role in the digestion of fish and in controlling the microbial content taken in with food via water.

Many bioindicators are used to determine the effects of xenobiotics in the aquatic environment, such as heat shock proteins (HSP) (Rangaswamy et al. 2024). Heat shock proteins are a large group of stress proteins found in all living organisms. HSP proteins are classified as HSP20, HSP40, HSP60, HSP70, HSP90, and HSP100 according to their molecular weights. HSPs are involved in the refolding of damaged proteins and the regulation of molecular processes under stress conditions. HSP70 expression in fish populations has been shown to exhibit an adaptive strategy against heat, salinity stress, pollutant exposure, and pathogen infection (Jeyachandran et al. 2023; Oksala et al. 2014; Oguz and Kaval Oguz, 2020; Zarei et al. 2024). When HSP70 was examined immunohistochemically in the gills, liver, and digestive tract, it was labelled more intensely in the post-spawning samples than in the pre-spawning groups (Figs. 13, 14, and 15). HSP70 observed in the tissues may indicate that spawning migration was a serious stress factor for pearl mullets. An increase in HSP70 levels was determined during migration in Sockeye Salmon (*Oncorhynchus nerka*) (Carey et al. 2019), supporting the present data.

## Conclusion

The present study demonstrated that the spawning migration of Pearl mullet induces substantial physiological, biochemical, and histological adjustments associated with reproduction, osmoregulation, and stress adaptation. Although pre- and post-spawning individuals exhibited similar lengths, a marked reduction in body weight and gonadosomatic index in post-spawning fish confirmed energy depletion and reproductive output. The observed increase in ATPase activities, particularly Na⁺/K⁺- and Ca^2^⁺-ATPases in the gill and kidney, highlights enhanced osmoregulatory activity necessary for re-establishing ionic balance after migration between freshwater and the saline-alkaline Lake Van. Concurrently, elevated antioxidant enzyme activities in post-spawning fish reflected oxidative stress resulting from intensive metabolic activity, tissue remodelling, and environmental transitions. Likewise, Fe distribution patterns also shifted, with higher Fe concentrations in the kidney pre-spawning and elevated levels in the gill, liver, and muscle post-spawning, indicating tissue-specific energy and oxygen demands across migration and recovery phases. The stability of acetylcholinesterase activity in the brain and muscle demonstrated the prioritisation of neural and muscular integrity despite systemic metabolic stress. Significant reductions in hepatic and muscular glucose and lipid levels further confirmed heavy energy utilisation during spawning migration, while histological changes in the gills and liver—such as lamellar fusion, increased mucus cells, and reduced glycogen content—revealed adaptive responses to environmental and physiological stressors. Finally, the elevated expression of HSP70 proteins across tissues underscored cellular stress and protective adaptation. Overall, these integrated responses emphasise the energetic and physiological complexity of pearl mullet migration and reproduction, highlighting the species’ remarkable resilience to environmental and metabolic challenges.

## Data Availability

Data in this manuscript will be shared upon request.
